# Association between oral candidiasis and low CD4+ count among HIV positive patients in Hoima Regional Referral Hospital

**DOI:** 10.1186/1472-6831-14-143

**Published:** 2014-11-28

**Authors:** Martina Nanteza, Jayne B Tusiime, Joan Kalyango, Arabat Kasangaki

**Affiliations:** Clinical Epidemiology Unit, Makerere University, Kampala, Uganda; School of Public Health, Makerere University, Kampala, Uganda; Department of Pharmacy, Makerere University, Kampala, Uganda; Department of Dentistry, Makerere University, P.O. Box 33019, Kampala, Uganda

**Keywords:** Oral candidiasis, Low CD4+ count, HIV positive patients

## Abstract

**Background:**

The aim of this study was to determine the prevalence of Human Immune Virus (HIV) related oral lesions and their association with Cluster of Differentiation 4 (CD4^+^) count among treatment naïve HIV positive patients.

**Methods:**

This was a descriptive and analytical cross sectional study. Participants were 346 treatment naïve HIV positive adult patients. These were consecutively recruited from Hoima Regional Referral hospital between March and April 2012. Data collection involved interviews, oral examinations and laboratory analysis.

**Results:**

A total of 168(48.6%) participants had oral lesions. The four commonest lesions were oral candidiasis (24.9%, CI = 20.6-29.7%), melanotic hyperpigmentation (17.3%, CI = 13.7-21.7%), kaposi sarcoma (9.3%, CI = 6.6-12.8%) and Oral Hairy Leukoplakia (OHL) (5.5%, CI = 3.5-8.4%). There was significant association between oral candidiasis and immunosuppression measured as CD4+ less than 350 cells/mm^3^ (OR = 2.69, CI = 1.608-4.502, p < 0.001). Oral candidiasis was the only oral lesion significantly predictive of immunosuppression (OR = 2.56, CI = 1.52-4.30, p < 0.001) with a Positive Predictive Value (PPV) of 48.2%, Negative Predictive Value (NPV) of 74.3%, 38.1% sensitivity and specificity of 81.4%.

**Conclusion:**

Oral candidiasis can be considered as a marker for immunesuppression, making routine oral examinations essential in the management of HIV positive patients.

## Background

HIV is a major global health problem. Sub-Saharan Africa continues to bear an inordinate share of the global HIV burden with 23 million people living with HIV/AIDS residing in this region [1]. Current HIV prevalence rates in Uganda stand at 7.4% [2] Among the HIV-associated infections, oral lesions have been recognized as prominent features since the beginning of the epidemic and continue to be important. Approximately 40-50% of people who are HIV-positive have been reported to have oral fungal, bacterial or viral infections, which often occur early in the course of HIV infection [3]. The occurrence of these oral lesions in HIV infection reflects the immune status of the patient with many being associated with reduced CD4+ T lymphocyte cell count. Because of this, they can be used as entry or end-points in therapy and vaccine trials as well as in staging and classification systems [4, 5]. They are thus not only important for their morbidity and mortality but also for their diagnostic value in monitoring the immune status of the patient.

In resource constrained settings, where routine access to immunological monitoring is limited, oral lesions have been suggested as useful aids that are complimentary to regular CD4+ count assessment [6]. In such settings therefore, monitoring is typically clinical and immunological,mainly as a result of financial and infrastructure constraints [7, 8]. There are cardinal lesions that are strongly associated with HIV and used internationally in the disease staging. The prevalence of such lesions amongst HIV positive individuals varies from region to region. Agwu et al. [9] found a prevalence of 71% in south western Uganda, while Tirwome et al. [10] recorded a 72% prevalence among HIV patients in several TASO clinics. Given that disease patterns change over time, and differ according to region studied its vital to know the current prevalence rates in Hoima. Information regarding association between the oral lesions and immunesuppression among HIV positive patients in Uganda is lacking. The aim of this study therefore was to highlight the prevalence of oral lesions among HIV positive patients, in the long run expressing the oral disease burden among HIV patients. The relationship between these lesions and the immune status of the patients was assessed in order to show how, if coupled with immunological monitoring, patients in resource limited settings can have the disease properly staged, classified and managed.

## Methods

### Participants and study setting

This was a descriptive and analytical cross-sectional study carried out from March to April 2012, in which quantitative data collection methods were used. It was carried out among consenting treatment naïve HIV positive male and female patients aged 18 years and above attending Hoima Regional Referral hospital HIV clinic. Patients who were too ill to participate in the interviews and oral examinations were excluded. Participants (n = 346) were consecutively selected as they presented to the clinic. Using an interviewer administered, standard and pre-tested questionnaire, information on socio-demographic characteristics; socio-behavioral factors; experience with oral lesions, and their consequences was collected. Information about history of systemic comorbidities, history of previous HIV oral lesions, and prescribed medication was obtained from the patient’s medical records.

### Investigations

Oral examinations were carried out by a dental surgeon, who was blinded to the clinical staging and CD4+ count results of the patients. Patients were examined while seated in a chair and in a well illuminated room. The extra oral and perioral areas were examined first, followed by intraoral tissues for any abnormalities. For better exploration of the mouth, periodontal probes, dental explorers and dental mirrors were used. Diagnosis of the oral lesions was made using European Community (EC) clearing house guidelines for presumptive diagnosis of oral lesions [11]. According to these guidelines, the lesions are classified into 3 groups: those strongly associated with HIV infection; those less commonly associated with HIV infection; and those seen in, but not indicative of HIV infection.

Lesions diagnosed were recorded into the World Health Organization (WHO) recording form for oral lesions associated with HIV infection. The inner and buccal surfaces of the lower incisors and the first two molars were assessed for presence of plaque or calculus. These were the lower incisors and the first two upper molars, whose inner and buccal surfaces were respectively examined. A photo atlas was developed to document the lesions diagnosed during the study. The clinical staging of new patients was done by the medical doctors in the HIV clinic.

For the regular patients, the clinical staging was retrieved from the patient’s records. The blood samples were obtained on the same day as the oral examinations and their results were recorded onto each participant’s questionnaire.

### Data management and quality control

Pretested questionnaires were used in data collection and were checked daily for consistency and completeness before the data was entered into the computer. Translation of the questionnaire into Runyakitara was done to cater for participants not well conversant with English. Data was entered in Epi data version 3.1, edited and cleaned and then exported to STATA version 9 for analysis.

### Data analysis

Descriptive analysis was used to summarize the participants’ characteristics using medians, percentages, frequencies and interquartile ranges. Odds ratios at 95% confidence intervals were used to measure association between each of the predictor variables and presence of oral lesions. Chi-square test with level of significance at 0.05 was used to test the significance of the association between presence of oral lesions and the predictor variables. CD4^+^ was categorized into CD4^+^ <350 cells/mm^3^ and CD4^+^ ≥350 cells/mm^3^, with immune suppression being defined as CD4^+^ < 350 cells/mm^3^[12]. Positive and negative predictive values of the most common lesions for CD4^+^ <350 cells/mm^3^ were computed. The sensitivity and specificity of the commonest lesions to diagnose immune suppression of <350 cells/mm3 were determined. Variables with p-value less than 0.2 at bivariate analysis were considered for inclusion in the multiple logistic regression models, with presence of oral lesions as the outcome. With CD4+ as the main predictor, other predictors were assessed for confounding and interaction.

### Ethical considerations

Permission to carry out the study was sought from Makerere University School of Medicine Research and Ethics Committee, the Uganda National Council of Science and Technology and Hoima hospital authorities. Written informed consent was obtained from the participants, and confidentiality was assured by concealing the patients’ names and using identification numbers on the questionnaires. Participants diagnosed with different oral lesions were treated in Hoima hospital and others who required further management were referred to the relevant health units.

## Results

Of the 346 consenting participants, 265(76.6%) were females and 81(23.4%) males (Table 1). The median age in this study group was 33 years (IQR, 25–41). Of the participants, 124(35.8%) had a history of regular alcohol consumption and 25(7.2%) had a history of regular tobacco smoking. About 24 (7%) of the respondents said they did not regularly brush their teeth, while 79(22.8%) reported brushing more than once daily. More than half of the respondents (53.2%, n = 184) reported never having gone for a dental checkup, and 154 (44.5%) reported going only when in discomfort. Most respondents had CD4+ count greater than 350 cells/mm^3^ (88.1%, n = 296), and majority of the respondents belonged to WHO stage two, (73.0%, n = 252).

**Table 1:** **Sociodemographic, sociobehavioral, dentobehavioral and clinical characteristics of 346 participants from Hoima Regional Referral Hospital**

Characteristics	Number	Proportion (%)
**Sex**		
Male	81	23.4
Female	265	76.6
**Smoking**		
Yes	25	7.2
No	321	92.8
**Alcohol consumption**		
Yes	124	35.8
No	222	64.2
**Brushing frequency**		
Not often	24	6.9
Once daily	243	70.2
More than once daily	79	22.8
**Dental visits**		
Never	184	53.2
Once a year	6	1.7
Every six months	2	0.6
When in discomfort	154	44.5
CD4* (n = 336)		
<350	105	31.3
≥350	231	68.7
**WHO stage**		
I	75	21.7
II	252	72.8
III	19	5.5
IV	0	0.0

*CD4+ count values for 10 subjects missing.

HIV associated oral lesions were observed in 48.6% (CI = 43.3-53.8%) (n = 168) respondents. Among the 346 respondents, the four most common lesions were oral candidiasis, melanotic hyperpigmentation, Kaposi sarcoma and OHL, in that order, as shown in Table 2 below. Oral candidiasis was present in 86 (24.9%) of the respondents, with the pseudomembranous subtype being the most prevalent and present among 42 respondents (12%). Melanotic hyperpigmentation was observed among 60 patients (17.3%, CI = 13.7-21.7%), whereas kaposi sarcoma was diagnosed among 32 participants (9.3% CI = 6.6-12.8%). Oral hairy leukoplakia was the least prevalent at 5.5% (n = 19, CI = 3.5-8.4%).

**Table 2:** **Prevalence of oral lesions among 346 Hoima hospital respondents**

Oral lesion	Frequency	Percentage	95% CI
Any oral lesion	168	48.6	43.3-53.8
Oral candidiasis	86	24.9	20.6-29.7
Pseudo membranous candidiasis	42	12.1	9.1-16.0
Erythematous candidiasis	29	8.4	5.9-11.8
Angular cheilitis	15	4.3	2.7-7.0
Oral hairy leukoplakia	19	5.5	3.5-8.4
Kaposi sarcoma	32	9.3	6.6-12.8
Melanotic hyperpigmentation	60	17.3	13.7-21.7
Linear gingival erythema	7	2.0	0.9-4.1
Herpes simplex	6	1.7	0.8-3.7
Recurrent apthous ulcers	5	1.5	0.6-3.3
Xerostomia	5	1.5	0.6-3.3
Salivary gland enlargement	13	3.8	2.2-6.3
Rampant caries	15	4.3	2.7-7.0
Calculus/plaque deposits	18	5.2	3.3-8.1

At bivariate analysis, CD4+ count of less than 350 cells/mm3, was found to be significantly associated with presence of oral candidiasis (OR = 2.691, p < 0.001,CI = 1.608-4.502). In the multivariate model, CD4+ count, problems with oral functions and WHO stage were significantly associated with the presence of oral candidiasis as shown in Table 3.

**Table 3:** **Results of multivariate analysis of factors associated with the presence of oral candidiasis**

Variable	OR	p-value	95% CI
**CD4+**	0.997	<0.001	0.995-0.999
**Problem with oral functions**			
None	1.000		
Chewing	7.389	<0.001	3.391-16.102
**WHO stage**			
Stage 1	1.000		
Stage 3	3.803	0.025	1.182-12.240

In the multivariate model, after analysis, we did not find any confounding by any of the dependent variables, and neither was there any interaction between them. Candidiasis was the only significant predictor of immune suppression measured as CD4 < 350 cells/mm^3^, (OR = 2.56, 1.52-4.30, p < 0.001), (Table 4) The PPV of OHL, kaposi sarcoma, melanotic hyperpigmentation and candidiasis were 47.4%, 41.9%, 32.2% and 48.2% respectively. The NPV of OHL, kaposi sarcoma, melanotic hyperpigmentation, and candidiasis were 69.7%, 69.8%, 68.9%, and 74.3% respectively. The sensitivity values were 8.6%, 12.4%,18.1% and 38.1% for OHL, kaposi sarcoma, melanotic hyperpigmentation and candidiasis respectively. Values for specificity were 95.7%, 92.2%, 82.7% and 81.4% for OHL, kaposi sarcoma, melanotic hyperpigmentation and candidiasis respectively.

**Table 4:** **Association of oral lesions with immune suppression (CD4 < 350 cells/mm** ^**3**^ **)**

Oral lesion	OR	95% CI	p-value	PPV	NPV	Sensitivity	Specificity
OHL	1.79	0.68-4.67	0.238	47.4	69.7	8.6	95.7
Kaposi sarcoma	1.48	0.67-3.27	0.334	41.9	69.8	12.4	92.2
Melanotic hyper pigmentation	0.96	0.51-1.81	0.896	32.2	68.9	18.1	82.7
Candidiasis	2.56	1.52-4.30	<0.001	48.2	74.3	38.1	81.4

## Discussion

### Prevalence of oral lesions

Since the beginning of AIDS epidemic, developing countries have experienced difficulties in implementing appropriate, inexpensive, and efficient HIV laboratory diagnostic techniques to aid in the epidemiological assessment and control of HIV infection. Studies have been conducted elsewhere for association of oral manifestations with absolute CD4 lymphocyte counts [13–15]. However, on doing a comprehensive literature search, there were no Ugandan studies for association of oral manifestations with CD4+ count. Hence an attempt was made in this study towards the correlation of oral manifestations with CD4+ count.

About five in every ten HIV patients in this study had oral lesions. The prevalence of oral lesions found in this study population was in agreement with the documented range of 30–80% seen in developing countries [16–19] and similar to findings from another study in Uganda [17]. It is however relatively lower than previously reported results from other studies done in different parts of Uganda where prevalence rates as high as 73.2% and 72% were found [9, 10]. This discrepancy is probably due to the fact that there has been increased support from government and donor agencies, and better strategies for management of HIV over the years.

Oral candidiasis was the commonest lesion observed in this study population, agreeing with findings by Bodhade et al. (39.3%), Kardpon et al. (55%), Adrogubangba et al. (33.3%) and Allan et al. (17.7%) [12, 15, 20, 21]. The pseudomembranous subtype was the commonest, just as was observed in south western Uganda (Table 2). Melanotic hyperpigmentation, although classified as a lesion less commonly associated with HIV, was the second most prevalent in this study. As the study group comprised of treatment naïve patients, the probability that this high prevalence was due to effects of antiretroviral therapy is very small. From their records no participants had previously been staged as stage four patients though surprisingly, Kaposi sarcoma which designates an individual to WHO stage four of HIV disease, was found in 32 (9.3%) of the participants. This exposes the discrepancies that occur in staging, and ultimately in management of HIV patients. Such discrepancies are more pronounced in resource limited settings where access to routine immunological monitoring is limited. Oral hairy leukoplakia had a low prevalence in this study, (5.5%, n = 19) probably due to the fact that definitive diagnosis requires demonstration of Epstein- Barr virus, which wasn’t conducted in this study and might have been a source of potential misclassification.

### Oral candidiasis in the prognosis of the disease

Findings showed that individuals under WHO stage three are almost 4 times as likely as individuals in stage one to develop oral candidiasis (Table 3). Oral candidiasis is currently being used to stage patients as stage three under the WHO clinical staging system, making these findings scientifically sound. In this study, HIV positive patients with difficulty in chewing are seven times more likely to have oral candidiasis than those without any difficulty in chewing. When a comprehensive dental history is taken, it is possible to elicit such symptoms, which when followed with an oral examination will aid in the identification and diagnosis of oral lesions. The oral lesions are in turn used to diagnose and monitor immunesuppression.

### Association between oral lesions and CD4+ count less than 350 cells/mm3

Oral candidiasis was the only lesion found to have a significant association with CD4+ count of less than 350 cells/mm3, (OR = 2.691, p < 0.001, CI = 1.608–4.502). Thus in this study, immunosuppressed individuals were almost thrice as likely to have oral candidiasis as their immune competent counterparts. In addition, oral candidiasis was the only lesion significantly predictive of immunesuppression in this study (Table 4). The findings imply that individuals with oral candidiasis are thrice as likely to be immunosuppressed as those without candidiasis. Therefore, oral candidiasis can be used as an index for immunosuppression depicted by CD4+ less than 350 cells in this study population.

These findings can be projected to other resource constrained settings, which makes routine oral examinations an essential part of management of HIV patients in these settings.

PPVs for oral manifestations to CD4+ less than 350 cells/mm3 were in a low range from 32.2% for melanotic hyperpigmentation to 48.2% for candidiasis (Table 4). Thus, with oral candidiasis as a test, forty eight (48) out of a hundred (100) HIV patients diagnosed as beingimmunosuppressed will truly be immunosuppressed. This value is much lower than that from studies by Patton et al. (60.3%) and Glick et al. (69.9%) [22, 23] owing to the low prevalence of immunosuppression in this study. The range for NPV was from 68.9% for melanotic hyperpigmentation to 74.3% for oral candidiasis Because the prevalence of immunosuppression in this population was low, the high NPV value of 74.3% for oral candidiasis makes it a not very informative test as regards to diagnosing the immunosuppression. Oral hairy leukoplakia had the highest specificity, of 95.7%. This means that when used as a test for immunesuppression, the likelihood for the occurrence of false positive results is low. As is already the practice, oral hairy leukoplakia is being utilized in patient staging. What these results emphasize is the importance of oral examinations during routine patient management.

Oral candidiasis had the highest sensitivity of 38.1%. Thus if oral candidiasis is used as a test, the proportion of individuals that would be correctly classified as immunosuppressed would be 68%, making candidiasis a fairly good test for the diagnosis of immunosuppression in the study population. Generally, the presence of oral candidiasis should serve to herald the possibility of a compromised immunity amongst HIV patients, thus making oral examinations vital and beneficial towards the management of the patients.

## Limitations, conclusions and recommendations

Diagnosis of the oral lesions was solely based on clinical findings, and no definitive diagnostic tests were performed. This might have introduced measurement bias into the study, in the form of non differential misclassification bias. However, the effect of this is likely to be minimal, as the diagnosis of the oral lesions followed an internationally accepted and recognized standard set of guidelines, the EC Clearing House guidelines. In addition, the significant odds ratios seen are actually true effect measures.

The study indicates that almost half in every one hundred study participants had an oral lesion, alone or in combination with other types, which is a moderately high rate.

The study demonstrated the possibility of oral candidiasis as an index to predict immunosuppression in resource limited settings with no access to regular CD4+ count monitoring. However to incorporate it as an index, certain regional parameters and research studies that employ rigorous research methodologies need to be done. There is a need to compare the results of various cross-sectional studies, and more longitudinal studies are required to formulate oral lesion indices that can help as markers for HIV-related immune suppression.
